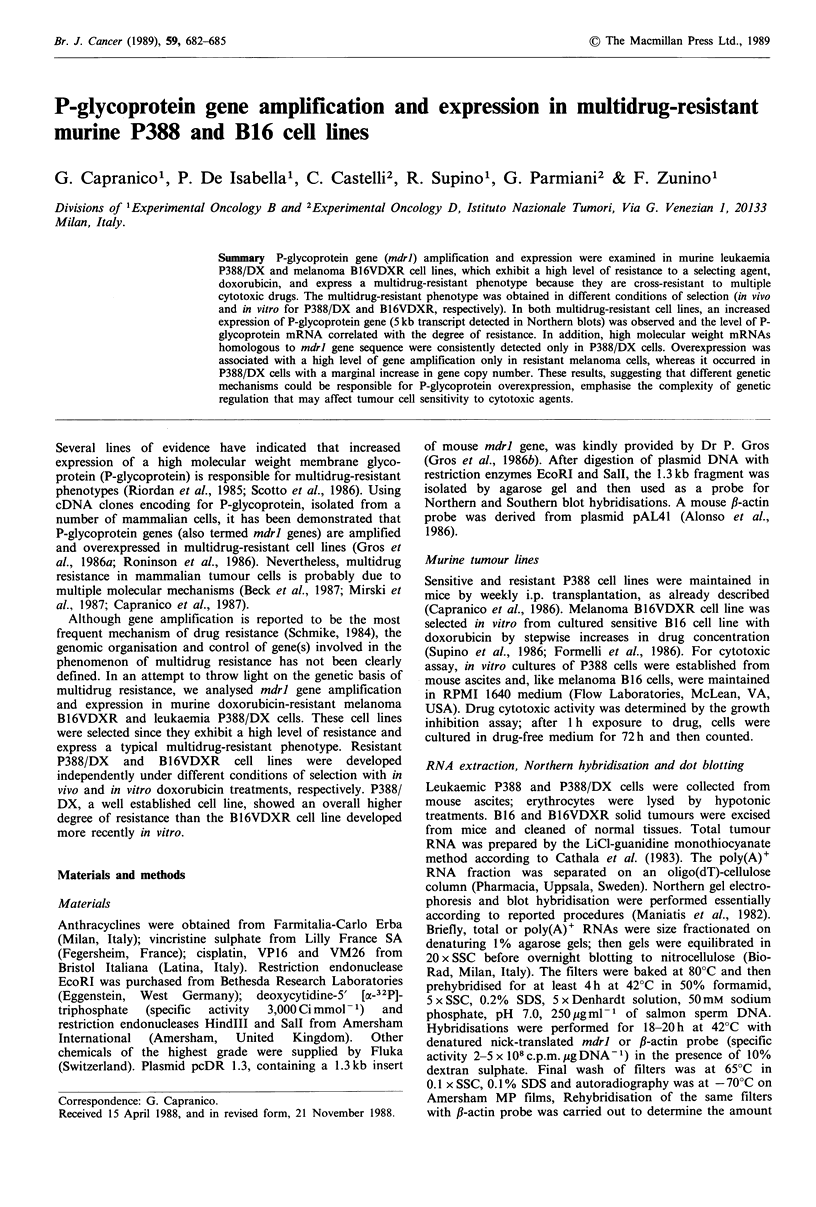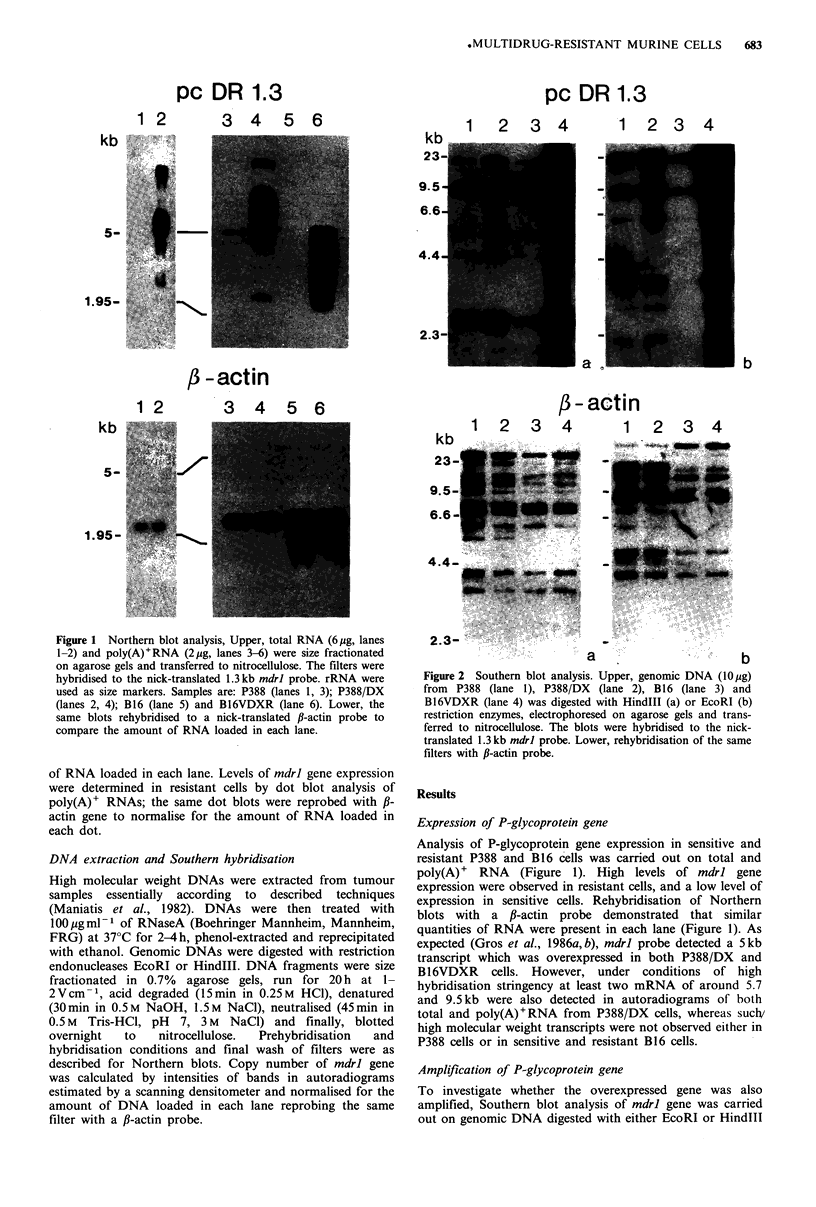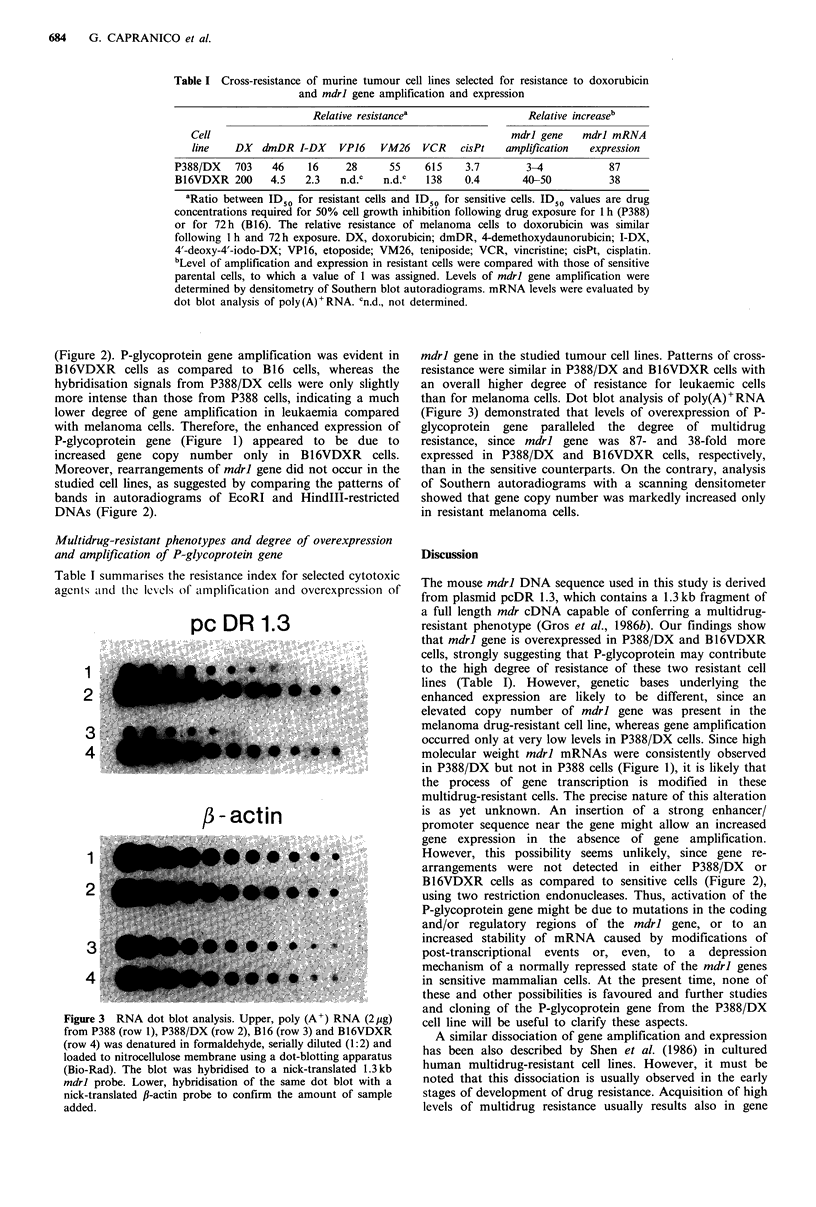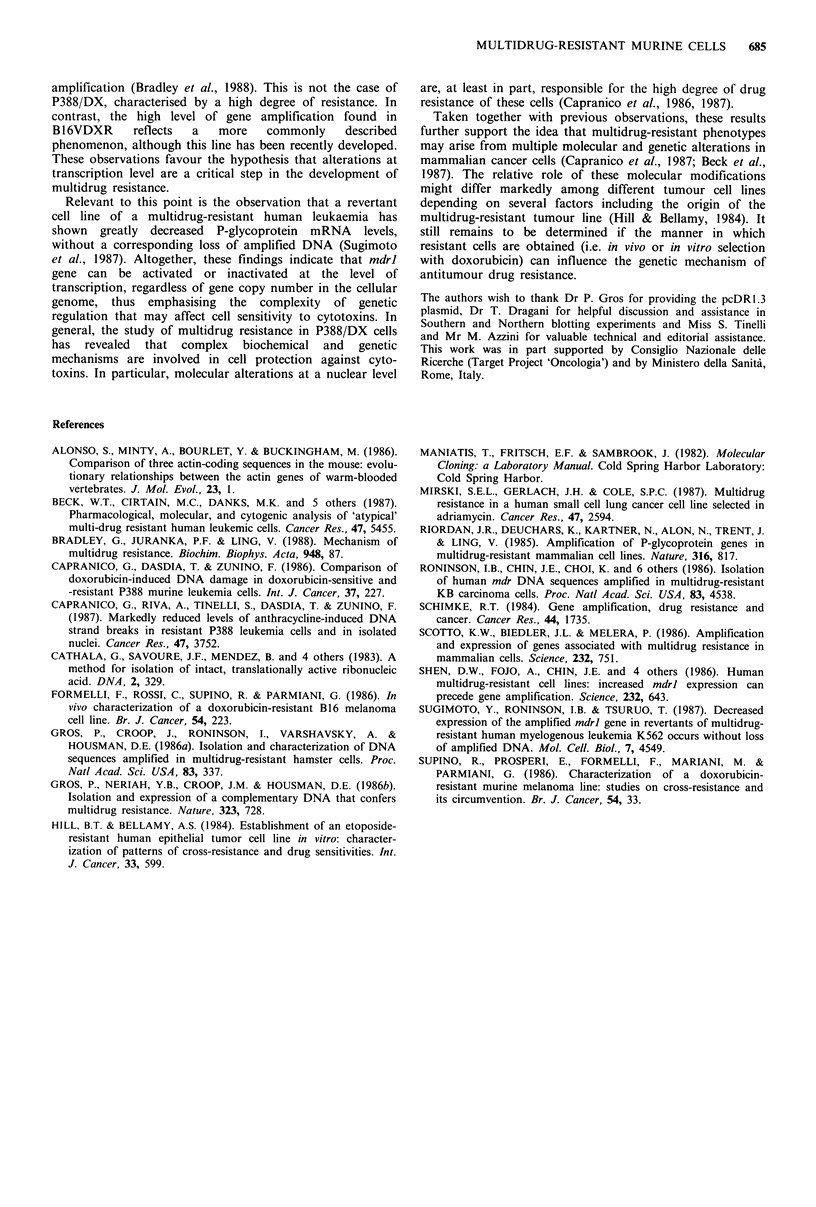# P-glycoprotein gene amplification and expression in multidrug-resistant murine P388 and B16 cell lines.

**DOI:** 10.1038/bjc.1989.141

**Published:** 1989-05

**Authors:** G. Capranico, P. De Isabella, C. Castelli, R. Supino, G. Parmiani, F. Zunino

**Affiliations:** Division of Experimental Oncology B, Istituto Nazionale Tumori, Milan, Italy.

## Abstract

**Images:**


*Br. J. Cancer* (1989), **59**, 682–685 © The Macmillan Press Ltd., 1989

### P-glycoprotein gene amplification and expression in multidrug-resistant murine P388 and B16 cell lines

G. Capranico[1], P. De Isabella[1], C. Castelli[2], R. Supino[1], G. Parmiani[2] & F. Zunino[1]

*Divisions of [1]Experimental Oncology B and [2]Experimental Oncology D, Istituto Nazionale Tumori, Via G. Venezian 1, 20133 Milan, Italy.*

**Summary** P-glycoprotein gene (*mdr1*) amplification and expression were examined in murine leukaemia P388/DX and melanoma B16VDXR cell lines, which exhibit a high level of resistance to a selecting agent, doxorubicin, and express a multidrug-resistant phenotype because they are cross-resistant to multiple cytotoxic drugs. The multidrug-resistant phenotype was obtained in different conditions of selection (*in vivo* and *in vitro* for P388/DX and B16VDXR, respectively). In both multidrug-resistant cell lines, an increased expression of P-glycoprotein gene (5 kb transcript detected in Northern blots) was observed and the level of P-glycoprotein mRNA correlated with the degree of resistance. In addition, high molecular weight mRNAs homologous to *mdr1* gene sequence were consistently detected only in P388/DX cells. Overexpression was associated with a high level of gene amplification only in resistant melanoma cells, whereas it occurred in P388/DX cells with a marginal increase in gene copy number. These results, suggesting that different genetic mechanisms could be responsible for P-glycoprotein overexpression, emphasise the complexity of genetic regulation that may affect tumour cell sensitivity to cytotoxic agents.

Several lines of evidence have indicated that increased expression of a high molecular weight membrane glycoprotein (P-glycoprotein) is responsible for multidrug-resistant phenotypes (Riordan *et al.*, 1985; Scotto *et al.*, 1986). Using cDNA clones encoding for P-glycoprotein, isolated from a number of mammalian cells, it has been demonstrated that P-glycoprotein genes (also termed *mdr1* genes) are amplified and overexpressed in multidrug-resistant cell lines (Gros *et al.*, 1986*a*; Roninson *et al.*, 1986). Nevertheless, multidrug resistance in mammalian tumour cells is probably due to multiple molecular mechanisms (Beck *et al.*, 1987; Mirski *et al.*, 1987; Capranico *et al.*, 1987).

Although gene amplification is reported to be the most frequent mechanism of drug resistance (Schmike, 1984), the genomic organisation and control of gene(s) involved in the phenomenon of multidrug resistance has not been clearly defined. In an attempt to throw light on the genetic basis of multidrug resistance, we analysed *mdr1* gene amplification and expression in murine doxorubicin-resistant melanoma B16VDXR and leukaemia P388/DX cells. These cell lines were selected since they exhibit a high level of resistance and express a typical multidrug-resistant phenotype. Resistant P388/DX and B16VDXR cell lines were developed independently under different conditions of selection with *in vivo* and *in vitro* doxorubicin treatments, respectively. P388/DX, a well established cell line, showed an overall higher degree of resistance than the B16VDXR cell line developed more recently *in vitro*.

### Materials and methods

### *Materials*

Anthracyclines were obtained from Farmitalia-Carlo Erba (Milan, Italy); vincristine sulphate from Lilly France SA (Fegersheim, France); cisplatin, VP16 and VM26 from Bristol Italiana (Latina, Italy). Restriction endonuclease EcoRI was purchased from Bethesda Research Laboratories (Eggenstein, West Germany); deoxycytidine-5′ [$\alpha$-$^{32}$P]-triphosphate (specific activity 3,000 Ci mmol$^{-1}$) and restriction endonucleases HindIII and SalI from Amersham International (Amersham, United Kingdom). Other chemicals of the highest grade were supplied by Fluka (Switzerland). Plasmid pcDR 1.3, containing a 1.3 kb insert of mouse *mdr1* gene, was kindly provided by Dr P. Gros (Gros *et al.*, 1986*b*). After digestion of plasmid DNA with restriction enzymes EcoRI and SalI, the 1.3 kb fragment was isolated by agarose gel and then used as a probe for Northern and Southern blot hybridisations. A mouse $\beta$-actin probe was derived from plasmid pAL41 (Alonso *et al.*, 1986).

Correspondence: G. Capranico.
Received 15 April 1988, and in revised form, 21 November 1988.

### *Murine tumour lines*

Sensitive and resistant P388 cell lines were maintained in mice by weekly i.p. transplantation, as already described (Capranico *et al.*, 1986). Melanoma B16VDXR cell line was selected *in vitro* from cultured sensitive B16 cell line with doxorubicin by stepwise increases in drug concentration (Supino *et al.*, 1986; Formelli *et al.*, 1986). For cytotoxic assay, *in vitro* cultures of P388 cells were established from mouse ascites and, like melanoma B16 cells, were maintained in RPMI 1640 medium (Flow Laboratories, McLean, VA, USA). Drug cytotoxic activity was determined by the growth inhibition assay; after 1 h exposure to drug, cells were cultured in drug-free medium for 72 h and then counted.

### *RNA extraction, Northern hybridisation and dot blotting*

Leukaemic P388 and P388/DX cells were collected from mouse ascites; erythrocytes were lysed by hypotonic treatments. B16 and B16VDXR solid tumours were excised from mice and cleaned of normal tissues. Total tumour RNA was prepared by the LiCl-guanidine monothiocyanate method according to Cathala *et al.* (1983). The poly(A)$^+$ RNA fraction was separated on an oligo(dT)-cellulose column (Pharmacia, Uppsala, Sweden). Northern gel electrophoresis and blot hybridisation were performed essentially according to reported procedures (Maniatis *et al.*, 1982). Briefly, total or poly(A)$^+$ RNAs were size fractionated on denaturing 1% agarose gels; then gels were equilibrated in 20 × SSC before overnight blotting to nitrocellulose (Bio-Rad, Milan, Italy). The filters were baked at 80°C and then prehybridised for at least 4 h at 42°C in 50% formamid, 5 × SSC, 0.2% SDS, 5 × Denhardt solution, 50 mM sodium phosphate, pH 7.0, 250 $\mu$g ml$^{-1}$ of salmon sperm DNA. Hybridisations were performed for 18–20 h at 42°C with denatured nick-translated *mdr1* or $\beta$-actin probe (specific activity 2–5 × 10$^8$ c.p.m. $\mu$g DNA$^{-1}$) in the presence of 10% dextran sulphate. Final wash of filters was at 65°C in 0.1 × SSC, 0.1% SDS and autoradiography was at −70°C on Amersham MP films, Rehybridisation of the same filters with $\beta$-actin probe was carried out to determine the amount

**Figure 1** Northern blot analysis, Upper, total RNA (6 μg, lanes 1–2) and poly(A)$^+$RNA (2 μg, lanes 3–6) were size fractionated on agarose gels and transferred to nitrocellulose. The filters were hybridised to the nick-translated 1.3 kb *mdr1* probe. rRNA were used as size markers. Samples are: P388 (lanes 1, 3); P388/DX (lanes 2, 4); B16 (lane 5) and B16VDXR (lane 6). Lower, the same blots rehybridised to a nick-translated β-actin probe to compare the amount of RNA loaded in each lane.

**Figure 2** Southern blot analysis. Upper, genomic DNA (10 μg) from P388 (lane 1), P388/DX (lane 2), B16 (lane 3) and B16VDXR (lane 4) was digested with HindIII (a) or EcoRI (b) restriction enzymes, electrophoresed on agarose gels and transferred to nitrocellulose. The blots were hybridised to the nick-translated 1.3 kb *mdr1* probe. Lower, rehybridisation of the same filters with β-actin probe.

of RNA loaded in each lane. Levels of *mdr1* gene expression were determined in resistant cells by dot blot analysis of poly(A)$^+$ RNAs; the same dot blots were reprobed with β-actin gene to normalise for the amount of RNA loaded in each dot.

### *DNA extraction and Southern hybridisation*

High molecular weight DNAs were extracted from tumour samples essentially according to described techniques (Maniatis *et al.*, 1982). DNAs were then treated with 100 $\mu g\,ml^{-1}$ of RNaseA (Boehringer Mannheim, Mannheim, FRG) at 37°C for 2–4 h, phenol-extracted and reprecipitated with ethanol. Genomic DNAs were digested with restriction endonucleases EcoRI or HindIII. DNA fragments were size fractionated in 0.7% agarose gels, run for 20 h at 1–2 $V\,cm^{-1}$, acid degraded (15 min in 0.25 M HCl), denatured (30 min in 0.5 M NaOH, 1.5 M NaCl), neutralised (45 min in 0.5 M Tris-HCl, pH 7, 3 M NaCl) and finally, blotted overnight to nitrocellulose. Prehybridisation and hybridisation conditions and final wash of filters were as described for Northern blots. Copy number of *mdr1* gene was calculated by intensities of bands in autoradiograms estimated by a scanning densitometer and normalised for the amount of DNA loaded in each lane reprobing the same filter with a β-actin probe.

### Results

### *Expression of P-glycoprotein gene*

Analysis of P-glycoprotein gene expression in sensitive and resistant P388 and B16 cells was carried out on total and poly(A)$^+$ RNA (Figure 1). High levels of *mdr1* gene expression were observed in resistant cells, and a low level of expression in sensitive cells. Rehybridisation of Northern blots with a β-actin probe demonstrated that similar quantities of RNA were present in each lane (Figure 1). As expected (Gros *et al.*, 1986*a*, *b*), *mdr1* probe detected a 5 kb transcript which was overexpressed in both P388/DX and B16VDXR cells. However, under conditions of high hybridisation stringency at least two mRNA of around 5.7 and 9.5 kb were also detected in autoradiograms of both total and poly(A)$^+$RNA from P388/DX cells, whereas such high molecular weight transcripts were not observed either in P388 cells or in sensitive and resistant B16 cells.

### *Amplification of P-glycoprotein gene*

To investigate whether the overexpressed gene was also amplified, Southern blot analysis of *mdr1* gene was carried out on genomic DNA digested with either EcoRI or HindIII

**Table I** Cross-resistance of murine tumour cell lines selected for resistance to doxorubicin and *mdr1* gene amplification and expression

| Cell line | Relative resistance[a] DX | dmDR | I-DX | VP16 | VM26 | VCR | cisPt | Relative increase[b] mdr1 gene amplification | mdr1 mRNA expression |
|---|---|---|---|---|---|---|---|---|---|
| P388/DX | 703 | 46 | 16 | 28 | 55 | 615 | 3.7 | 3–4 | 87 |
| B16VDXR | 200 | 4.5 | 2.3 | n.d.[c] | n.d.[c] | 138 | 0.4 | 40–50 | 38 |

[a]Ratio between $ID_{50}$ for resistant cells and $ID_{50}$ for sensitive cells. $ID_{50}$ values are drug concentrations required for 50% cell growth inhibition following drug exposure for 1 h (P388) or for 72 h (B16). The relative resistance of melanoma cells to doxorubicin was similar following 1 h and 72 h exposure. DX, doxorubicin; dmDR, 4-demethoxydaunorubicin; I-DX, 4′-deoxy-4′-iodo-DX; VP16, etoposide; VM26, teniposide; VCR, vincristine; cisPt, cisplatin. [b]Level of amplification and expression in resistant cells were compared with those of sensitive parental cells, to which a value of 1 was assigned. Levels of *mdr1* gene amplification were determined by densitometry of Southern blot autoradiograms. mRNA levels were evaluated by dot blot analysis of poly(A)$^+$RNA. [c]n.d., not determined.

(Figure 2). P-glycoprotein gene amplification was evident in B16VDXR cells as compared to B16 cells, whereas the hybridisation signals from P388/DX cells were only slightly more intense than those from P388 cells, indicating a much lower degree of gene amplification in leukaemia compared with melanoma cells. Therefore, the enhanced expression of P-glycoprotein gene (Figure 1) appeared to be due to increased gene copy number only in B16VDXR cells. Moreover, rearrangements of *mdr1* gene did not occur in the studied cell lines, as suggested by comparing the patterns of bands in autoradiograms of EcoRI and HindIII-restricted DNAs (Figure 2).

*Multidrug-resistant phenotypes and degree of overexpression and amplification of P-glycoprotein gene*

Table I summarises the resistance index for selected cytotoxic agents and the levels of amplification and overexpression of *mdr1* gene in the studied tumour cell lines. Patterns of cross-resistance were similar in P388/DX and B16VDXR cells with an overall higher degree of resistance for leukaemic cells than for melanoma cells. Dot blot analysis of poly(A)$^+$RNA (Figure 3) demonstrated that levels of overexpression of P-glycoprotein gene paralleled the degree of multidrug resistance, since *mdr1* gene was 87- and 38-fold more expressed in P388/DX and B16VDXR cells, respectively, than in the sensitive counterparts. On the contrary, analysis of Southern autoradiograms with a scanning densitometer showed that gene copy number was markedly increased only in resistant melanoma cells.

**Figure 3** RNA dot blot analysis. Upper, poly (A$^+$) RNA (2 μg) from P388 (row 1), P388/DX (row 2), B16 (row 3) and B16VDXR (row 4) was denatured in formaldehyde, serially diluted (1:2) and loaded to nitrocellulose membrane using a dot-blotting apparatus (Bio-Rad). The blot was hybridised to a nick-translated 1.3 kb *mdr1* probe. Lower, hybridisation of the same dot blot with a nick-translated β-actin probe to confirm the amount of sample added.

### Discussion

The mouse *mdr1* DNA sequence used in this study is derived from plasmid pcDR 1.3, which contains a 1.3 kb fragment of a full length *mdr* cDNA capable of conferring a multidrug-resistant phenotype (Gros *et al.*, 1986*b*). Our findings show that *mdr1* gene is overexpressed in P388/DX and B16VDXR cells, strongly suggesting that P-glycoprotein may contribute to the high degree of resistance of these two resistant cell lines (Table I). However, genetic bases underlying the enhanced expression are likely to be different, since an elevated copy number of *mdr1* gene was present in the melanoma drug-resistant cell line, whereas gene amplification occurred only at very low levels in P388/DX cells. Since high molecular weight *mdr1* mRNAs were consistently observed in P388/DX but not in P388 cells (Figure 1), it is likely that the process of gene transcription is modified in these multidrug-resistant cells. The precise nature of this alteration is as yet unknown. An insertion of a strong enhancer/promoter sequence near the gene might allow an increased gene expression in the absence of gene amplification. However, this possibility seems unlikely, since gene re-arrangements were not detected in either P388/DX or B16VDXR cells as compared to sensitive cells (Figure 2), using two restriction endonucleases. Thus, activation of the P-glycoprotein gene might be due to mutations in the coding and/or regulatory regions of the *mdr1* gene, or to an increased stability of mRNA caused by modifications of post-transcriptional events or, even, to a depression mechanism of a normally repressed state of the *mdr1* genes in sensitive mammalian cells. At the present time, none of these and other possibilities is favoured and further studies and cloning of the P-glycoprotein gene from the P388/DX cell line will be useful to clarify these aspects.

A similar dissociation of gene amplification and expression has been also described by Shen *et al.* (1986) in cultured human multidrug-resistant cell lines. However, it must be noted that this dissociation is usually observed in the early stages of development of drug resistance. Acquisition of high levels of multidrug resistance usually results also in gene amplification (Bradley *et al.*, 1988). This is not the case of P388/DX, characterised by a high degree of resistance. In contrast, the high level of gene amplification found in B16VDXR reflects a more commonly described phenomenon, although this line has been recently developed. These observations favour the hypothesis that alterations at transcription level are a critical step in the development of multidrug resistance.

Relevant to this point is the observation that a revertant cell line of a multidrug-resistant human leukaemia has shown greatly decreased P-glycoprotein mRNA levels, without a corresponding loss of amplified DNA (Sugimoto *et al.*, 1987). Altogether, these findings indicate that *mdr1* gene can be activated or inactivated at the level of transcription, regardless of gene copy number in the cellular genome, thus emphasising the complexity of genetic regulation that may affect cell sensitivity to cytotoxins. In general, the study of multidrug resistance in P388/DX cells has revealed that complex biochemical and genetic mechanisms are involved in cell protection against cytotoxins. In particular, molecular alterations at a nuclear level are, at least in part, responsible for the high degree of drug resistance of these cells (Capranico *et al.*, 1986, 1987).

Taken together with previous observations, these results further support the idea that multidrug-resistant phenotypes may arise from multiple molecular and genetic alterations in mammalian cancer cells (Capranico *et al.*, 1987; Beck *et al.*, 1987). The relative role of these molecular modifications might differ markedly among different tumour cell lines depending on several factors including the origin of the multidrug-resistant tumour line (Hill & Bellamy, 1984). It still remains to be determined if the manner in which resistant cells are obtained (i.e. *in vivo* or *in vitro* selection with doxorubicin) can influence the genetic mechanism of antitumour drug resistance.

The authors wish to thank Dr P. Gros for providing the pcDR1.3 plasmid, Dr T. Dragani for helpful discussion and assistance in Southern and Northern blotting experiments and Miss S. Tinelli and Mr M. Azzini for valuable technical and editorial assistance. This work was in part supported by Consiglio Nazionale delle Ricerche (Target Project 'Oncologia') and by Ministero della Sanità, Rome, Italy.

### References

ALONSO, S., MINTY, A., BOURLET, Y. & BUCKINGHAM, M. (1986). Comparison of three actin-coding sequences in the mouse: evolutionary relationships between the actin genes of warm-blooded vertebrates. *J. Mol. Evol.*, **23**, 1.

BECK, W.T., CIRTAIN, M.C., DANKS, M.K. and 5 others (1987). Pharmacological, molecular, and cytogenic analysis of 'atypical' multi-drug resistant human leukemic cells. *Cancer Res.*, **47**, 5455.

BRADLEY, G., JURANKA, P.F. & LING, V. (1988). Mechanism of multidrug resistance. *Biochim. Biophys. Acta*, **948**, 87.

CAPRANICO, G., DASDIA, T. & ZUNINO, F. (1986). Comparison of doxorubicin-induced DNA damage in doxorubicin-sensitive and -resistant P388 murine leukemia cells. *Int. J. Cancer*, **37**, 227.

CAPRANICO, G., RIVA, A., TINELLI, S., DASDIA, T. & ZUNINO, F. (1987). Markedly reduced levels of anthracycline-induced DNA strand breaks in resistant P388 leukemia cells and in isolated nuclei. *Cancer Res.*, **47**, 3752.

CATHALA, G., SAVOURE, J.F., MENDEZ, B. and 4 others (1983). A method for isolation of intact, translationally active ribonucleic acid. *DNA*, **2**, 329.

FORMELLI, F., ROSSI, C., SUPINO, R. & PARMIANI, G. (1986). *In vivo* characterization of a doxorubicin-resistant B16 melanoma cell line. *Br. J. Cancer*, **54**, 223.

GROS, P., CROOP, J., RONINSON, I., VARSHAVSKY, A. & HOUSMAN, D.E. (1986*a*). Isolation and characterization of DNA sequences amplified in multidrug-resistant hamster cells. *Proc. Natl Acad. Sci. USA*, **83**, 337.

GROS, P., NERIAH, Y.B., CROOP, J.M. & HOUSMAN, D.E. (1986*b*). Isolation and expression of a complementary DNA that confers multidrug resistance. *Nature*, **323**, 728.

HILL, B.T. & BELLAMY, A.S. (1984). Establishment of an etoposide-resistant human epithelial tumor cell line *in vitro*: characterization of patterns of cross-resistance and drug sensitivities. *Int. J. Cancer*, **33**, 599.

MANIATIS, T., FRITSCH, E.F. & SAMBROOK, J. (1982). *Molecular Cloning: a Laboratory Manual*. Cold Spring Harbor Laboratory: Cold Spring Harbor.

MIRSKI, S.E.L., GERLACH, J.H. & COLE, S.P.C. (1987). Multidrug resistance in a human small cell lung cancer cell line selected in adriamycin. *Cancer Res.*, **47**, 2594.

RIORDAN, J.R., DEUCHARS, K., KARTNER, N., ALON, N., TRENT, J. & LING, V. (1985). Amplification of P-glycoprotein genes in multidrug-resistant mammalian cell lines. *Nature*, **316**, 817.

RONINSON, I.B., CHIN, J.E., CHOI, K. and 6 others (1986). Isolation of human *mdr* DNA sequences amplified in multidrug-resistant KB carcinoma cells. *Proc. Natl Acad. Sci. USA*, **83**, 4538.

SCHIMKE, R.T. (1984). Gene amplification, drug resistance and cancer. *Cancer Res.*, **44**, 1735.

SCOTTO, K.W., BIEDLER, J.L. & MELERA, P. (1986). Amplification and expression of genes associated with multidrug resistance in mammalian cells. *Science*, **232**, 751.

SHEN, D.W., FOJO, A., CHIN, J.E. and 4 others (1986). Human multidrug-resistant cell lines: increased *mdr1* expression can precede gene amplification. *Science*, **232**, 643.

SUGIMOTO, Y., RONINSON, I.B. & TSURUO, T. (1987). Decreased expression of the amplified *mdr1* gene in revertants of multidrug-resistant human myelogenous leukemia K562 occurs without loss of amplified DNA. *Mol. Cell. Biol.*, **7**, 4549.

SUPINO, R., PROSPERI, E., FORMELLI, F., MARIANI, M. & PARMIANI, G. (1986). Characterization of a doxorubicin-resistant murine melanoma line: studies on cross-resistance and its circumvention. *Br. J. Cancer*, **54**, 33.